# Integrating remote high-intensity interval training into multi-component obesity treatment for adolescents: Impacts on body composition, fitness, and lifestyle

**DOI:** 10.1016/j.obpill.2025.100176

**Published:** 2025-04-26

**Authors:** Fábio de Freitas, Mariana R. Zago, Maria Ângela Antônio, Maria Ângela Bellomo Brandão, António Videira-Silva

**Affiliations:** a Unicamp School of Medical Sciences, Department of Pediatrics, Child and Adolescent Health Graduate Program, Campinas, São Paulo, Brazil; b Unicamp School of Medical Sciences, Department of Pediatrics, Campinas, São Paulo, Brazil; c Clínica Universitária de Pediatria, Faculdade de Medicina, Universidade de Lisboa, Lisbon, Portugal; d CIDEFES (Research Centre in Sport, Physical Education, Exercise and Health), Universidade Lusófona, Lisbon, Portugal; e CIFI2D (Centre for Research, Training, Innovation, and Intervention in Sport), Universidade do Porto, Porto, Portugal

**Keywords:** Adolescent, Obesity, Exercise therapy, High-intensity interval training, Home care services

## Abstract

**Background:**

This study aimed to analyze the effects of a three-month remote High-Intensity Interval Training (HIIT) program as an adjunct to a multi-component clinical obesity treatment on body composition, physical fitness, movement behaviors, and nutritional habits in adolescents with obesity.

**Methods:**

This study was designed as a non-randomized controlled trial involving a total of 100 adolescents with obesity (BMI z-score ≥2), aged 12–17, divided into a control group (CG, receiving only standard care, i.e., medical and nutritional guidance, n = 50), and an experimental group (EG, exposed to a remote HIIT program four times/week (∼20 min/session) for 3 months, additionally to standard care, n = 50). Intervention effect was analyzed based on adherence (presence in ≥80 % of sessions). Anthropometrics, body composition, and physical fitness data were assessed at baseline and at the end of the intervention. Changes in body composition and physical fitness were the primary outcomes, while movement behaviors and nutritional habits were considered secondary outcomes.

**Results:**

Six participants were excluded from the analysis due to missing post-intervention assessments. Among the 44 (88 %) adolescents who completed the 3-month assessments, 28 were included in the adherents’ group (AG) and 16 in non-adherents (non-AG). BMI z-score significantly decreased over time (β = −0.08, p = 0.001), with the AG showing a more significant reduction than non-AG (β = −0.3, p < 0.001) and CG (β = −0.29, p < 0.001). Flexibility (β = 3.5, p < 0.001) and Core strength improved (β = 2.9, p = 0.002), with no differences between the AG and non-AG. Water consumption also increased (β = 0.2, p = 0.022), but only in the AG.

**Conclusion:**

The remote HIIT program was effective in improving body composition and physical fitness in adolescents with obesity. These findings highlight the potential of remote exercise interventions as a feasible and beneficial strategy within multi-component obesity treatments.

## Highlights

• Remote orientation may enhance access to exercise by removing transportation and time barriers.
• The HIIT program was feasible as a multi-component obesity treatment, improving body composition and fitness.
• The small changes in behavior and intake underscore the need for ongoing support to sustain habits.

## Graphical abstract

**Figure:**
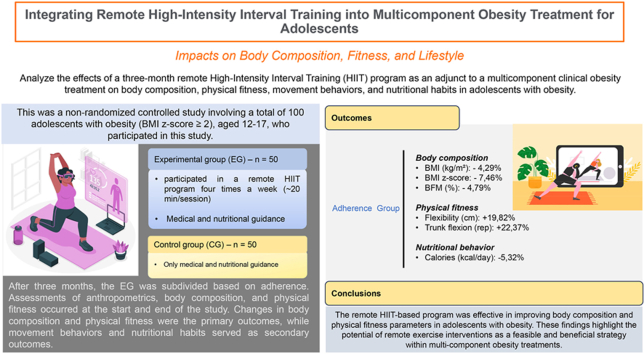

## 1 Introduction

Adolescence is a period of significant physical, physiological, and psychological changes [1]. Establishing healthy habits, including regular physical activity (PA), is essential during this period, as it contributes to the healthy development of the body and mind, preventing long-term negative consequences [2].

Recent evidence suggests an increase in physical inactivity in this population [3], which can lead to an increased incidence of obesity and other chronic non-communicable diseases (NCDs), which can extend into adulthood [4,5]. It is estimated that about 8 % of Brazilian children and adolescents are obese (BMI z-score ≥2) [6]. Despite the relatively low prevalence of pediatric obesity compared to most European countries, the prevalence of physical inactivity is high in Brazil, with around 75 % of children and adolescents not meeting PA guidelines (i.e., 60 min per day of moderate to vigorous PA) [7]. Even so, it is estimated that a 10 % reduction in the prevalence of physical inactivity would be associated with a decrease in NCDs and related hospitalization costs in Brazil [8,9].

Physical exercise (a subset of planned, structured, and repetitive PA designed to improve or maintain physical fitness) [10] has been recognized as one of the most relevant non-pharmacological strategies for treating pediatric obesity and NCDs [[11], [12], [13]]. However, face-to-face exercise-based programs can be limited by contextual barriers such as transportation costs, time constraints, and accessibility, which may hinder participation and reduce long-term adherence [14,15].

Remote exercise programs can overcome some of the barriers of in-person programs and represent an opportunity to improve pediatric obesity care [16,17]. Remote interventions may include the use of telecommunications or virtual technologies (eHealth and mHealth) such as electronic devices (e.g., activity trackers), active video games, mobile health apps, web-based platforms, and real-time video calls [18], which can be used alone or as a support strategy for face-to-face programs. Multi-component approaches involving in-person interactions and remote support may be more effective compared to single interventions [19].

In recent years, there has been a growing interest in the effectiveness of exercise in face-to-face programs as an option for treating obesity in adolescents [20,21]. High-intensity interval training (HIIT), a training protocol consisting of short but intense exercise sessions interspersed with brief periods of rest, has been touted as a safe and effective exercise protocol for treating obesity in adolescents [20], as it is associated with higher excess post-exercise oxygen consumption (EPOC) and adherence, compared to other exercise protocols [22]. However, the potential effect of HIIT may also depend on adherence [17].

We hypothesize that a remotely supervised exercise program utilizing a HIIT-based protocol as an additional component of a multi-component clinical treatment for pediatric obesity may provide an opportunity to enhance the treatment of obesity in adolescents. Therefore, this study aimed to analyze the effects of a three-month remote HIIT-based program as an adjunct to a multi-component clinical obesity treatment on body composition and physical fitness, including changes in movement behaviors and nutritional habits among adolescents with obesity.

## 2 Methods

### 2.1 Study design

This non-randomized controlled trial implemented a three-month remote HIIT-based program as an adjunct to a comprehensive clinical obesity treatment. Initially, 100 adolescents (58 % boys) were included and divided into two groups: one experimental group (EG) (n = 50, 30 % boys), which participated in four weekly HIIT sessions (≈20 min per session) and received support from a pediatrician and a nutritionist; and one control group (CG) (n = 50, 28 % boys), which received only standard care, i.e., professional counseling from a pediatrician and a nutritionist, without engaging in structured exercise sessions. CG counseling included discussion of potential limitations after clinical assessment, guidance on portion sizes, risks of ultra-processed food consumption, adequate water consumption, strategies for mindful eating, and counseling on physical activity, according to the Brazilian Society of Pediatrics [23] and National Dietary [24] and Physical Activity Guidelines [25]. No clinical appointments were held during the intervention (i.e., 3 months) for any groups.

After the intervention, participants in the experimental group were categorized based on adherence to the HIIT program. Those who attended at least 80 % of the sessions were classified as adherent (AG), while those with irregular participation but who completed the final assessments were considered non-adherent (non-AG). The CG remained unchanged throughout the study.

The primary outcomes involved changes in physical fitness and body composition. The secondary outcomes involved alterations in movement behavior and nutritional habits. Assessments were conducted at the baseline and after three months of intervention.

### 2.2 Participants

The study included adolescents with obesity (BMI z-score ≥2, for gender and age, according to the World Health Organization) [26], aged between 12 and 17 years, of both sexes, followed at the Child and Adolescent Obesity Outpatient Clinic between September 2021 and March 2023 were invited to participate.

#### 2.2.1 Exclusion criteria

The participants were excluded from the study if they had disabling orthopedic or neurological injuries, were unable to engage in regular PA or exercise, suffered from mental disorders, participated in another weight loss or PA program (except for Physical Education at school), or lacked an electronic device with a camera and internet access.

Medical experts assessed the clinical condition and cardiovascular risk of all participants prior to the exercise intervention to discuss any limitations to exercise (e.g., mechanical constraints, target heart rate). Participants' enrollment in the study was contingent on the assessment by these specialists.

### 2.3 Workout protocol

Participants engaged in a structured HIIT-based exercise protocol consisting of approximately 20-min sessions performed four times per week over three months, totaling 48 sessions.

Sessions included: 5 min warm-up (e.g., dynamic stretching, joint mobility exercises); the central section included 10 min of combining aerobic and resistance exercises (e.g., squat, butt kicker, toe touches, lunges, mountain climbers, standing abs twist, squat to side, lunges, squats, back bridge, planks, jumping jacks.), structured as a circuit training protocol, ≈4 sets of 40 s of exercise followed by 10 s of recovery between sets; and 5 min cool-down (e.g., low-intensity coordination exercises and isometric stretching).

All participants in the EG followed the same training protocol, which included a week of acclimatization. During this period, the intensity, difficulty, and duration of the exercises were progressively adjusted to minimize the risk of delayed-onset muscle soreness. The intensity of the sessions was regulated according to the participants' subjective perception of exertion [27].

#### 2.3.1 Synchronous online remote session

The exercise sessions were led by a qualified physical education professional using the Google Meet platform for real-time video calls. Participants were asked to keep their cameras on throughout the sessions to facilitate adjustments and corrections to their exercises. The sessions were scheduled for 9 a.m. and 5 p.m. to avoid conflicts with school routines.

### 2.4 Variables and instruments

#### 2.4.1 Anthropometry and body composition

Height was assessed with a fixed stadiometer (Sanny, ES2020) in the anthropometric position, without shoes, after the expiratory phase, and with an accuracy of 0.1 cm [28].

Body weight was measured with a mechanical anthropometric scale (Welmy, Brazil) with a precision of 0.1 kg. Participants were wearing as little clothing as possible, without shoes, and with the arms positioned alongside the body [28].

Waist circumference (WC) was measured with an inelastic tape measure (Sanny) after the expiratory phase without skin compression [28].

The Body Mass Index (BMI) was calculated as body weight in kilograms divided by the square of the height in meters [(BMI = weight (kg)/height2 (m)]. BMI z-score (BMIz) was calculated according to the WHO reference using the AnthroPlus calculator (version 1.0.4, WHO) [26].

Body composition was assessed by single-frequency bioelectrical bioimpedance (Quantum II, RJL Systems, USA) with participants in the supine position, wearing light clothing and without shoes or socks. Four self-adhesive electrodes were placed on the right foot (at the base of the middle finger and above the ankle joint line) and on the right hand (at the base of the middle finger and above the wrist joint line). The skin was cleaned with alcohol before the electrodes were placed [29]: total body fat mass percentage (BFM) and skeletal muscle mass percentage (SMM).

#### 2.4.2 Physical fitness assessment

Flexibility was assessed using the sit-and-reach test using the Wells bench (Sanny, Brazil). The participant sat on a mat with their legs straight, knees locked, and the soles of their feet against the equipment. With hands overlapping and palms down, the participant stretched along the measurement line, holding the position for 1–2 s. The distance was recorded in increments of 0.1 cm [30].

The trunk flexion test was used to assess localized muscle resistance. During the test, repetitions were not counted if the elbow did not touch the knee or the back did not touch the mat. The number of sit-ups performed in 1 min was recorded [30].

#### 2.4.3 Movement behaviors

The International Physical Activity Questionnaire—short form (IPAQ-SF) was used to classify each participant's initial PA level [31]. The analysis used the daily mean of the time stationary and the weekly means of Light PA (LPA), moderate PA (MPA), vigorous PA (VPA), and moderate-vigorous PA (MVPA ​= ​MPA ​+ ​VPA).

#### 2.4.4 Nutritional information

Eating behavior was assessed using the 24-h recall method, incorporating both detailed records and standardized interviews [32].

### 2.5 Statistical analysis

Data were analyzed using the IBM SPSS statistical program (version 28.0, IBM, New York, USA). The normality of the distribution was tested using the Shapiro-Wilk test and confirmed by the observation of the histogram. The mean and standard deviation were used for data with normal distribution, while the median and interquartile range were used for asymmetric data. The differences between the groups were analyzed using the independent *t*-test and the Mann-Whitney test for regular and asymmetric data, respectively.

Quantile regression mixed-effects models were employed to analyze longitudinal changes in the measured variables. Results were presented as unstandardized beta coefficients (β) and 95 % confidence intervals (CI). The effect of the intervention over time (3 months) was analyzed, using the control group as reference category.

A p-value ≤ 0.05 was considered statistically significant.

## 3 Results

### 3.1 Baseline characteristics

At baseline in EG, 50 participants with a mean age of 14.1 (±1.8) years, while the CG had a mean age of 13.9 (±1.2) years were included. There was no significant difference between the groups in terms of age (Table 1).

**Table 1:** Baseline characteristics of participants.

*Variables*	Experimental (*n=50*)	Control (*n=50*)	*p-value*^a^
	**Mean ± SD**		
Age (years)	14.1 ± 1.8	13.9 ± 1.2	0.978
***Anthropometry***			
Weight (kg)	104.4 ± 28.1	94.4 ± 26.1	0.075
Height (cm)	165.8 ± 9.1	165.2 ± 9.4	0.732
WC (cm)	103.1 ± 13.9	97.1 ± 14.6	0.038
***Body composition***			
BMI (kg/m2)	37.6 ± 8.1	34.2 ± 7.3	0.027
BMI z-score	3.67 ± 1.9	3.14 ± 1.04	0.020
BFM (%)	37.0 ± 6.4	37.5 ± 5.4	0.658
***Movement behavior***			
LPA (min/day)	28.0 ± 22.8	8.3 ± 9.4	< 0.001
MVPA (min/day)	26.6 ± 26.4	10.1 ± 17.0	< 0.001

Mean ± SD: standard deviation.

WC: waist circumference; BMI: body mass index; BFM: body fat mass; SMM: skeletal muscle mass: LPA: light physical activity; MVPA: moderate-vigorous physical activity.

a Differences between groups analyzed with the independent *t*-test and the Mann-Whitney *U* test.

On average, WC was higher in the EG (103.1 ± 13.9 cm) compared to the CG (97.1 ± 14.6 cm, p = 0.038). BMI was also significantly higher in the EG than in the CG (37.6 ± 8.1 kg/m^2^ vs. 34.2 ± 7.3 kg/m^2^, p = 0.027), as was the BMI z-score (3.67 ± 1.9 vs. 3.14 ± 1.5, p = 0.048) (Table 1).

Regarding PA levels, in mean, the EG engaged in significantly more LPA (28.0 ± 22.8 min/day vs. 8.3 ± 9.4 min/day, p < 0.001) and MVPA (26.6 ± 26.4 min/day vs. 10.1 ± 17.0 min/day, p < 0.001) compared to the CG (Table 1).

#### 3.1.1 Compliance

After the intervention, participants in the EG were categorized based on adherence to the remote HIIT-based program. Six participants were excluded from the analysis due to missing post-intervention assessments. Among the 44 (88 %) adolescents who completed the 3-month assessments, a total of 28 participants attended at least 80 % of the sessions, being classified as adherent group (AG), and 16 as non-adherent (non-AG). The CG remained unchanged throughout the study.

No unintended effects were reported during or as a consequence of the intervention.

Table 2 presents the mean changes in body composition, physical fitness, movement behavior, and nutritional habits from the baseline to three months post-intervention, categorized by group.

**Table 2:** Baseline and three-month group differences in body composition, physical fitness, movement, and nutritional behavior.

*Variables*	Assessment					
	Adherence (n = 28)		Non - Adherence (n = 16)		Control (n = 50)	
***Body composition***	***Baseline***	***month 3***	***Baseline***	***month 3***	***Baseline***	***month 3***
BMI (kg/m^2^)	39.6 ± 9.0	37.9 ± 8.9	34.3 ± 5.5	34.9 ± 5.7	34.2 ± 7.3	34.6 ± 7.4
BMI z-score	3.89 ± 1.17	3.60 ± 1.16	3.24 ± 0.69	3.28 ± 0.73	3.14 ± 1.04	3.15 ± 1.06
BFM (%)	37.6 ± 7.2	37.1 ± 7.4	34.9 ± 5.5	35.2 ± 5.2	37.5 ± 5.4	37.3 ± 5.5
SMM (%)	62.4 ± 7.2	62.8 ± 7.5	65.1 ± 5.5	64.8 ± 5.1	–	–
***Physical fitness***						
Flexibility (cm)	22.2 ± 8.6	26.6 ± 8.7	22.4 ± 9.1	23.4 ± 10.6	–	–
Trunk flexion (rep)	15.2 ± 11.0	18.6 ± 10.3	14.9 ± 7.7	15.7 ± 9.3	–	–
***Movement behavior***						
Screen time (h/day)	5.0 ± 2.5	5.1 ± 2.7	4.9 ± 2.9	4.8 ± 3.0	4.6 ± 2.8	4.9 ± 2.7
Sleep duration (h/day)	8.4 ± 1.1	8.4 ± 1.2	7.8 ± 1.2	8.0 ± 1.4	8.3 ± 1,7	8.0 ± 1.4
***Nutritional behavior***						
Water intake (L/day)	1.3 ± 0.9	1.5 ± 0.7	1.6 ± 0.7	1.6 ± 0.7	1.4 ± 1.0	1.6 ± 0.9
Sugary beverages (ml/day)	160.3 ± 144.4	168.1 ± 147.8	256.3 ± 258.1	293.3 ± 234.4	322.6 ± 198.0	330.4 ± 227.3
Calories (kcal/day)	1607.2 ± 609.1	1521.7 ± 477.5	1629.3 ± 349.4	1643.6 ± 468.0	1724.3 ± 419.3	1750.6 ± 559.0

Mean ± SD: standard deviation.

BFM: body fat mass; BMI: body mass index; BFM: body fat mass; SMM: skeletal muscle mass.

### 3.2 Effects on body composition

According to quantile regression mixed-effects models, the BMI z-score decreased significantly over time (β = −0.08, 95 % CI: −0.12 to −0.03, p = 0.001). The AG experienced a more pronounced reduction compared to the non-AG (β = −0.3, 95 % CI: −0.4 to −0.2, p < 0.001) and the CG (β = −0.29, 95 % CI: −0.4 to −0.2, p < 0.001) (Table 3).

**Table 3:** Comparative longitudinal analysis in body composition, physical fitness, movement, and nutritional behavior over time between groups.

*Variables*	Intervention^a^	*p-value*	Adherence x non-adherence	*p-value*	Adherence x Control	*p-value*
***Body composition***	***β (95 % CI)***		***β (95 % CI)***		***β (95 % CI)***	
BMI (kg/m^2^)	−0.2 (-0.5, 0.1)	0.252	**−2.0 (-2.8, - 1.3)**	**<** 0**.001**	**−2.0 (-2.6, -1.5)**	**<** 0**.001**
BMI z-score	**−0.08 (-0.12, - 0.03)**	0**.001**	**−0.3 (-0.4, - 0.2)**	**<** 0**.001**	**−0.29 (-0.4, - 0.2)**	**<** 0**.001**
BFM (%)	−0.2 (-0.5, 0.1)	0.194	−0.6 (−2.4, 1.1)	0.456	−0.3 (−1.5, 0.9)	0.591
SMM (%)	0.2 (-0.8, 1.1)	0.726	0.5 (-1.1, 2.2)	0.520	–	–
***Physical fitness***						
Flexibility (cm)	**3.5 (2.3, 4.8)**	**<** 0**.001**	2.6 (-0.1, 5.4)	0.061	–	
Trunk flexion (rep)	**2.9 (1.1, 4.7)**	0**.002**	1.6 (-1.9, 5.0)	0.369	–	
***Movement behavior***						
Screen time (h/day)	−0.2 (-0.2, 0.6)	0.257	0.1 (-1.1, 1.3)	0.880	−0.2 (-1.0, 0.7)	0.683
Sleep duration (h/day)	−0.1 (-0.4, 0.2)	0.476	−0.4 (-0.9, 0.2)	0.204	0.3 (-0.2, 0.7)	0.279
***Nutritional behavior***						
Water intake (L/day)	**0.2 (0.0, 0.3)**	0**.022**	0.3 (-0.1, 0.6)	0.128	−0.1 (-0.5, 0.3)	0.619
Sugary beverages (ml/day)	−12.2 (-59.5, 35.1)	0.607	−47.2 (−179.4, 85.0)	0.477	61.5 (−47.9, 171.0)	0.264
Calories (kcal/day)	−39.1 (-164.6, 86.4)	0.534	−88.8 (-348.8, 171.2)	0.496	−54.7 (-346.7, 237.3)	0.708

Data presented β (95 % CI); CI: confidence interval.

Bold β indicates a significant interaction (p ≤ .05) group-by-time interaction; β: expected median value; βs adjusted for sex and age.

BFM: body fat mass; BMI: body mass index; BFM: body fat mass; SMM: skeletal muscle mass.

a Quantile regression mixed models were used to analyze over time; Intervention (time: 3 months); Control group as a reference category.

### 3.3 Effects on physical fitness

According to quantile regression mixed-effects models, flexibility increased significantly over time (β = 3.5, 95 % CI: 2.3 to 4.8, p < 0.001). The AG showed greater flexibility than the non-AG (β = 2.6, 95 % CI: −0.1 to 5.4, p = 0.061), but this difference was not statistically significant (Table 3).

Trunk flexion repetitions also increased significantly (β = 2.9, 95 % CI: 1.1 to 4.7, p = 0.002). However, no significant differences emerged between the AG and non-AG (β = 1.6, 95 % CI: −1.9 to 5.0, p = 0.369). (Table 3).

### 3.4 Effects on movement behaviors

According to quantile regression mixed-effects models, screen time and sleep duration remained unchanged across the groups, with no statistically significant differences (Table 3).

### 3.5 Effects on nutritional behavior

According to quantile regression mixed-effects models, higher water intake was associated with an increase in (β = 0.2, 95 % CI: 0.0 to 0.3, p = 0.022). In contrast, calorie intake remained similar across the groups, showing no significant differences (Table 3).

## 4 Discussion

This study examined the effects of a three-month remote HIIT-based program, which was included as an additional component of the multi-component clinical obesity treatment for adolescents. The results suggest that the intervention with the remote HIIT-based program promoted improvements in the primary outcomes. Although the changes in movement behaviors were not statistically significant, the observed trends indicated potential benefits, providing a positive perspective for the future of pediatric obesity management. These results support the inclusion of remote HIIT-based as a complementary strategy in clinical obesity treatment programs.

The effects of the remote HIIT-based program showed a reduction in BMI and BMI z-score among adolescents with obesity who adhered to it. Comparing this group with the non-AG and CG highlighted these differences, emphasizing that achieving health benefits depends on adherence [33]. Supporting our findings, Vandoni et al. (2022) also reported a decrease in BMI Z-score among adolescents following a 12-week online HIIT program [34]. Conversely, the lack of significant effects on BFM and SMM in the AG suggests that the duration of the intervention may have been inadequate to induce meaningful changes in these variables.

Our results differ from previous studies that indicated the effects of HIIT on reducing body fat and increasing muscle mass in pediatric populations with obesity [32,33]. This difference can be attributed to variations in the methodologies of the studies, the intervention duration, the frequency and intensity of training, the types of exercises used, and the characteristics of the sample [21]

The results from a study of online physical education classes conducted by Lee et al. (2021) indicated significant improvements in muscle strength and balance. However, the analyzed groups did not achieve relevant improvements in flexibility and core resistance (trunk flexion) [37]. In contrast, our findings indicated improvements in these variables over time in the AG compared to the non-AG. Besides the mechanical and postural benefits, these improvements might be linked to indirect metabolic gains, such as increased basal energy expenditure and enhanced mobilization of energy substrates [22]. These results highlight the relevance of choosing the type of training since different approaches can impact other components of physical fitness in a specific way [36].

Physical exercise is a fundamental strategy for boosting energy expenditure and is widely regarded as a sustainable method for preventing and treating obesity [13]. Among the various training techniques, HIIT is notable for its effectiveness in enhancing post-exercise oxygen consumption and promoting fat oxidation[38], thereby expanding its metabolic benefits [35]. Furthermore, HIIT can elevate the resting metabolic rate, aiding in the reduction of central adiposity [39], [40]. Consequently, high-intensity, short-duration protocols hold promise for maximizing the impact of exercise on managing childhood obesity, encouraging and motivating further research and implementation [20]. Based on the gathered evidence, the intervention protocol utilized in this study was tailored and modified to address the characteristics and needs of the target population, with the goal of increasing energy expenditure, improving body composition, and optimizing metabolic outcomes [21], [39].

Although all groups received follow-up care from pediatricians and dietitians as part of the outpatient health care plan, changes in dietary patterns were modest. The literature indicates that more intense exercise can lead to a 6–11 % reduction in daily caloric intake, resulting in a negative energy balance [41]. In the present study, the group participating in the remote HIIT-based program experienced an average decrease of 5.32 % in caloric intake in three months. However, this reduction did not achieve statistical significance. According to the Transtheoretical Model of Behavior Change, adopting new habits is a gradual process [42]. For instance, incorporating an exercise routine might trigger a trend of reduced caloric intake. However, this transition happens at different rates, leading to distinct short-term changes [43].

On the other hand, the study identified -six strengths: (i) the simple, HIIT-based program at home (approximately 20 min per session), four times a week, maybe a viable strategy when integrated as an additional component in multi-component clinical treatments for obesity; (ii) sessions consisting of a combination of aerobic and resistance exercises using body weight were chosen based on relevant literature, making the protocol practical and replicable; (iii) participants did not need to purchase materials; (iv) there was online and synchronous monitoring that included feedback, support, and progress tracking; (v) the reduction of costs and travel time, along with the flexibility of schedules; and (vi) importantly, no injuries or physical complications were reported during the intervention, reinforcing the safety and feasibility of the remote HIIT sessions.

## 5 Limitations

Despite the promising findings, some limitations should be considered when interpreting the results of this study. For instance, adherence to the program was 56 % among participants, while 32 % did not adhere but attended the final evaluations. The main barriers included maladjusted family environments (such as violence, loss of a sibling, and attempted matricide), low motivation, lack of family support, or the perception that exercise was unnecessary. Some parents, however, actively participated in the exercise sessions or motivated their sons, reinforcing adherence. The lack of information on parental involvement may be considered a limitation since parent participation may lead to better obesity-related outcomes in children and adolescents with obesity [44].

Other possible limitations include the non-randomized design, which may have introduced selection bias, reducing the ability to draw causal inferences; and subjectivity in the report, both in questionnaire responses and in perceived exercise intensity, that may also have introduced bias, potentially influenced by social desirability. Additionally, the single-center setting, small sample size, and short intervention period limit the generalizability of the findings and may be insufficient to assess long-term effects.

Moreover, the requirement for internet access and camera-enabled devices could have introduced socioeconomic bias by excluding adolescents from more vulnerable backgrounds. Furthermore, the inclusion of the HIIT-based intervention within a broader multidisciplinary model does not allow the attribution of effects exclusively to the intervention. This limitation was, however, partially addressed by the inclusion of a CG that was not exposed to the intervention.

Despite these limitations, the study offers novel insights by evaluating a HIIT-based intervention embedded in a real-world, multidisciplinary healthcare setting for pediatric obesity treatment. The findings highlight the potential of this approach and the importance of future research, particularly longitudinal and multicenter studies, to assess the sustainability of outcomes, focusing on fostering autonomy and long-term engagement in physical activity.

## 6 Conclusion

The remote HIIT-based program emerged as a viable and safe alternative to complement the clinical management of obesity in adolescents, with suggested improvements in body composition and physical fitness, while facilitating exercise supervision and guidance through real-time video calls. Additionally, the program influenced movement behaviors and nutritional habits, reinforcing its role as an effective adjunct in multi-component obesity treatment.

## Author contributions

Conceptualization, FF, and AVS.; data curation, FF, and AVS.; formal analysis, FF.; funding acquisition, MARGM, and MAB; investigation, MRZ, and FF; methodology, FF, AVS and MAB; project administration, MAB; resources, MAB; supervision, AVS and MAB.; validation, AVS and MAB; visualization, MRZ, MARGM and MAB.; writing—original draft, FF.; writing—review and editing, FF, AVS, MRZ, MARGM, and MAB. All authors have read and agreed to the published version of the manuscript.

## Ethics

This study was approved by the Research Ethics Committee of the State University of Campinas under opinion number 4.940.045, following the norms established by Resolution 466/2012 of the National Health Council. All participants and their guardians who agreed to participate signed a free and informed consent form.

## Artificial intelligence (AI)

During the preparation of this work the authors did not use AI assisted technologies.

## Declaration of competing interest

The authors declare the following financial interests/personal relationships which may be considered as potential competing interests: Fabio Freitas reports financial support was provided by Coordination of Higher Education Personnel Improvement. If there are other authors, they declare that they have no known competing financial interests or personal relationships that could have appeared to influence the work reported in this paper.
